# Using theory of change in child health service interventions: a scoping review protocol

**DOI:** 10.12688/wellcomeopenres.17553.1

**Published:** 2022-01-28

**Authors:** Benjamin Jones, Shobhana Nagraj, Mike English

**Affiliations:** 1 Oxford Centre for Global Health Research, Nuffield Department of Medicine, University of Oxford, Oxford, UK; 2 KEMRI-Wellcome Trust Research Programme, KEMRI-Wellcome Trust, Nairobi, Kenya

**Keywords:** evaluation, intervention development, paediatrics, program theory, quality improvement, implementation science, theory of change

## Abstract

**Background: **The objective of this scoping review is to map the evidence of how child health service interventions use their theory of change. A theory of change is a hypothesis of *how* and *why* an intervention is intended to bring about change. It can be used as a program design, implementation, and evaluation tool. This scoping review will provide an overview of the evidence base for, and identify the way in which, theories of change in child health service interventions are defined, rationalised, developed, presented, and refined.

**Methods:** The inclusion criteria for this scoping review is any child health service intervention globally, that describes their theory of change or theory of change development process. Relevant exclusions include: logic models or logic frameworks that do not meet this review’s definition of theory of change, systematic reviews, behavioural change interventions that target patient’s behaviour, school-based interventions, and maternal health interventions not related to child health outcomes. This scoping review will follow the Joanna Briggs Institute Reviewer’s manual. Relevant publications will be first searched on selected electronic databases and grey literature. A search strategy will be developed. The search will be limited to articles written in the English language. Results of the search will be curated using Endnote and duplicates removed. Results will be imported to Rayyan. The inclusion criteria will be applied during the process of title and abstract screening, by two independent reviewers and disagreements resolved by a third independent reviewer. Full-texts will have the inclusion criteria applied via the same reviewer process. Data relevant to the research sub-questions will be extracted, analysed, charted and discussed.

**Ethics and dissemination: **Ethical approval is not required for this review as we will make use of already published data. We aim to publish the findings of our review in a peer-reviewed journal.

## Introduction

Health service interventions are interventions targeting the access to, and the use, costs, quality, delivery, organisation, financing, and outcomes of health care services such as hospitals and primary healthcare services ( Institute of Medicine Committee on Health Services Research, 1994; Lohr & Steinwachs, 2002; Steinwachs, 1991). An intervention is any organised activity, program, project, or initiative, that is supported by resources and established with the purpose of enacting change. This scoping review will focus on chid health service interventions, defined as any such intervention with a target population of children aged 0–19 years old. Health service interventions are often complex by nature, involving multiple stakeholders, diverse objectives, and are dependent in context. A theory of change (ToC) is a tool utilised by some health service interventions to navigate this complexity.

A theory of change (ToC) is a theory or hypothesis of *how* and *why* an intervention is intended to bring about change ( Center for Theory of Change, 2021; Connell & Kubisch, 1998; De Silva *et al*., 2014; Weiss, 1995). When a ToC is reported in internal or external organisation publications, it is an articulation of the hypothesised pathways that lead to change. It presents the cumulative study of an intervention's assumptions, activities, mechanisms, measurement indicators, outcomes, and context, as well as the linkages between these various components ( Connell & Kubisch, 1998).

A ToC can be presented in narrative form or illustrated visually using ToC diagrams. These diagrams are referred to by a number of different terms, depending on subtleties in their form and contents. These terms include, but are not limited to: logic models, logic frameworks, logframes, outcome hierarchies, and result chains. Inappropriate use of these terms can create confusion amongst the ToC literature ( Weiss, 1997b). It is therefore important in this scoping review to explicitly outline what is meant and not meant by the term theory of change. For example, perhaps the most common term used incorrectly as a ToC synonym is logic model. De Silva *et al*. (2014) outline that logic models, strictly speaking, are more simple, linear, and rigid diagrams than true ToC diagrams, that do not outline assumptions, measurement indicators or describe a consideration of the intervention’s causal relationships. ‘Indicators’ are the ToC terminology for the specific measurable marker used to determine if each activity performed in the proposed causal pathway and associated outcomes in a ToC, are achieved. This data can be either qualitative or quantitative. Indicators should include the population group related to the activity or outcome and the threshold that is the minimum accepted achievement of the outcome ( Taplin & Clark, 2012). An example a ToC diagram and a logic model have been included as *Extended data* to aid understanding ( Jones, 2022).

The terms ToC, and the synonymous theory-based evaluation, are generally accepted to have been popularised in the 1990s, stemming from decades of work in program evaluation and program theory ( Connell & Kubisch, 1998; Connell *et al*., 1995; Weiss, 1995; Weiss, 1997b). They have been used in many fields, including health, education, business, community development and social welfare ( Center for Theory of Change, 2021; De Silva *et al*., 2014; Weiss, 1997b). Despite this seemingly broad application base, the value that a ToC brings to an intervention needs to be considered if more organisations are to invest the resources required to articulate and present a comprehensive ToC. Even as early as 1987, Huey-Tsyh Chen and Peter Rossi argued for the internal and external validity of a theory based evaluation approach ( Chen & Rossi, 1987). Then in 1997, Carol Weiss, one of the architects behind the development of ToC articulation, noted that the literature was “replete with paeans to the value of the theory based approach” to program evaluation ( Weiss, 1997a). That is, a number of studies speak to the benefits of the ToC development process such as helping to engage stakeholders, clarify focus, enhance connection to the intervention, and engage team thinking around the process that underpins interventions. Furthermore, these studies report the value that a ToC provides once completed, including providing a roadmap for the intervention, a clear anchor for evaluation, a base in which to refine thinking and monitor the intervention as context changes overtime, scale-up utility, and identify intentional and unintentional consequences ( Anderson, 2006; Blamey & Mackenzie, 2007; Connell & Kubisch, 1998; De Silva *et al*., 2014; Rogers *et al*., 2004; Weiss, 1997a; Weiss, 1997b). However, it is important to consider that there is a selection bias of papers in the literature that present beneficial ToC articulations, rather than those that failed and were stopped short. Some issues with ToC identified in the literature include: the confusion around definition, that they are reductionist models of more open systems that give a false sense of control to the intervention implementers, they are not developed with enough rigour, and provide an excuse not to adapt when context changes ( Connell & Kubisch, 1998; De Silva *et al*., 2014; Weiss, 1995; Weiss, 1997a). Overall, this scoping review will aim to answer the question: how are ToCs for child health service interventions developed, utilised, and refined? It will also aim to answer the following research sub-questions:

1. How do these studies define ToC?
2. What is the rationale for the ToC being developed?
3. What is the process of development of the ToC?
4. Who is involved in the development of the ToC?
5. At what stage in the intervention are ToCs developed?
6. How are the ToCs presented in the literature?
7. In what way is the ToC used (purpose)?
8. Is the value of the ToC outlined, and if so what is it and the evidence supporting it?
9. How is the ToC refined overtime?

A preliminary search of MEDLINE, the Cochrane Database of Systematic Reviews and JBI Evidence Synthesis was conducted and no current or underway systematic reviews or scoping reviews on the topic were identified. Although no reviews were found, this initial search revealed a number of studies that may meet the eligibility criteria. For example, in 2016, Breuer *et al*. conducted a review of the use of ToC in *public health* interventions. This article acts as an example of a review of individual studies that describe how ToCs are developed and refined in healthcare. Another such paper, Kumar *et al*. (2020) uses a ToC to describe the service level interventions needed to be implemented to prevent retinopathy of prematurity and the associated indicators to monitor progress. Another, Makowiecka *et al*. (2019) established a common ToC for 61 unique maternal and newborn interventions, funded by a single philanthropic organisation. Finally, Hanson *et al*. (2019) developed a protocol of an evaluation study for a neonatal health project in India, which provides a detailed ToC and ToC development process attached as a specific appendix.

A scoping review was selected for this study as it will provide a mapping opportunity as well as an overview of the available research evidence; allowing for the identification of the way in which ToC in child health service interventions are defined, rationalised, developed, presented, and refined. This scoping review is intended to inform organisations working in the field of child health about how they may design and utilise their own ToC. Ultimately, the objective of this scoping review is to map the evidence how child health service interventions around the world develop, use, and refine their theory of change.

## Methods

The proposed scoping review will be conducted in accordance with the JBI methodology for scoping reviews ( Joanna Briggs Institute, 2015; Peters *et al*., 2020) and was also used for the development of this protocol.

### Eligibility criteria

***Participants.*** Health service interventions targeted at children and youth aged 0–19 years will be included. Health service interventions will be defined as interventions targeting the access to, and the use, costs, quality, delivery, organisation, financing, and outcomes of health care services. The term ‘intervention’ will be defined as any organised activity, program, project, or initiative, that is supported by resources and established with the purpose of enacting change. This scoping review will exclude studies in which the main target of the intervention was individual patients rather than the health service more broadly. For example, an intervention using behavioural change theory aiming to influence a patient’s cognition and behaviour would be excluded. However, if an intervention used behavioural change theory aiming to change the behaviour of staff members in order to more effectively deliver a healthcare service, this will be included. Settings outside healthcare facilities such as schools, will be excluded. Obstetric health service interventions that aim to improve maternal health outcomes rather than newborn health outcomes will also be excluded. Some health service interventions that overlap with public health interventions such as vaccination programs will be included in this scoping review.

***Concept.*** Interventions that discuss their ToC will be included. Specifically, papers will be included if they a) describe how a ToC was utilised throughout any stage of a child health service intervention such as design, implementation, or evaluation. Or b) describe the development process for a ToC planned to be used in a child health service intervention. A ToC will be defined as a theory or hypothesis of *how* and *why* an intervention is intended to bring about change. As highlighted by Breuer *et al*. (2016), it may be difficult to ascertain if a study has discussed a ToC as per this definition purely from the title and abstract alone. Therefore, during title and abstract screening a study will be included for full-text screening if it refers to the development of their own ToC, theory-based evaluation, theory informed evaluation, program theory, outcomes hierarchy, results chain logic model, or logic framework or, if the title/abstract include comments on the steps of *how,* or mechanisms behind *why,* an intervention worked. Then, during full-text screening, when a more thorough analysis of how these studies used the various terms above can be conducted, the specific definition of ToC above will be applied. The included studies should include a consideration of at least one of the following ToC components; assumptions, activities, mechanisms, measurement indicators, outcomes, and context, or the linkages between these various components.

***Sources.*** This scoping review will consider a number of different study designs including both quantitative, qualitative and mixed-methods study designs. From the grey literature, organisational e.g., private organisations and NGO’s as well as government ToC documents will be included. Systematic reviews will not be included. There will be no other limits of date, study design, or type of publication.

### Search strategy

The search strategy will aim to locate both published and unpublished studies. An initial limited search of MEDLINE was conducted to identify articles on the topic. The text words contained in the titles and abstracts of relevant articles, and the index terms used to describe the articles were used to develop full search strategies for each database (see *Extended data*). The search strategy, including all identified keywords and index terms, will be adapted for each included database and/or information source. Broadly, the database search will combine terms from the two themes: childhood age range and ToC. The reference list of all included sources of evidence will be screened for additional studies.

Studies published in English will be included. This is due to resourcing constraints within the research team. No publication year restrictions will be used in the search in order to gain an understanding of how different terminology has been used over time.

The databases to be searched include, MEDLINE, EMBASE, Global Health, WHO Global Index Medicus, CINAHL and SCOPUS. Sources of unpublished studies/ grey literature to be searched include the first 10 pages of a specific google search ‘Child* "theory of change" filetype:pdf’. The titles and then full-texts of these pdfs will then have the same criteria applied as the literature. The most recent search of these sources in formulating the search strategy was completed on 14 ^th^ December 2021.

### Evidence selection

This will be an iterative process whereby the literature will be searched and the search strategy refined. Following the search, all identified citations will be collated and uploaded into Endnote V20 and duplicates removed before being uploaded to Rayyan, an online review management software. Following a pilot test, titles and abstracts will then be screened by two or more independent reviewers for assessment against the inclusion criteria for the review. The inclusion and exclusion criteria were refined and agreed by all three researchers. As outlined in the introduction, there are a number of terms used to describe diagrams that are similar to, but often lack the depth of, a complete ToC. This presents a challenge during particularly the title and abstract screening stage, where a paper may not explicitly acknowledge an articulation of a ToC and label it as such. Therefore, careful analysis of the abstracts to identify if comments are made on the ‘how’ and ‘why’ an intervention has worked, will be critical when there is an absence of specific phrases such as; ToC, theory based evaluation, theory informed evaluation, logic model, logic framework, or program theory. This approach to study selection diverges from that of the review conducted by Breuer *et al*. (2016) who limited the criteria to needing the specific term ‘theory of change’ to be explicitly mentioned in the title or abstract order for the paper to meet the inclusion criteria.

Following this, potentially relevant sources will be retrieved in full. The full text of selected citations will be assessed in detail against the inclusion criteria by two or more independent reviewers. Reasons for exclusion of sources of evidence at full text that do not meet the inclusion criteria will be recorded and reported in the scoping review. Any disagreements that arise between the reviewers at each stage of the selection process will be resolved through discussion. The results of the search and the study inclusion process will be reported in full, in the final scoping review and presented in a Preferred Reporting Items for Systematic Reviews and Meta-analyses extension for scoping review (PRISMA-ScR) flow diagram.

### Data collection

Data will be extracted from papers included in the scoping review by two or more independent reviewers using a data extraction tool developed by all reviewers, to aid with consistency of which data will be extracted ( *see Extended data*). The data extracted will include specific details about the participants, concept, context, study methods and key findings relevant to the review questions. A draft extraction form is provided (see *Extended data*). The draft data extraction tool will be modified and revised as necessary during the process of extracting data from each included evidence source. Modifications will be detailed in the scoping review. Any disagreements that arise between the reviewers will be resolved through discussion, or with an additional reviewer(s). If appropriate, authors of papers will be contacted to request missing or additional data, where required.

***Data analysis and presentation.*** Data will be presented in table form. Furthermore, a narrative summary will accompany the tabulated information and will describe how the results relate. Data will be analysed using both qualitative and quantitative methods. Qualitative data analysis will involve all reviewers engaging in an in-depth discussion about the ToC data and describing the major concepts arising. Literature will be analysed to study location, type of health service intervention, and ToC definition (using a checklist of key ToC components such as assumptions, activities, mechanisms, measurement indicators, outcomes, context, and linkages), ToC presentation. Data extracted from the data extraction tool (see *Extended data*) will then be charted as per the JBI. Firstly, in the data charting process, a calibration exercise with the full team will be implemented. This will involve a random sample of 20 citations for title and abstract screening. A roundtable discussion will be had to clarify any issues. After the initial calibration exercise, one author (BJ) will chart the data independently and two authors (ME and SN) will verify the data for accuracy. Inconsistencies and disagreements will be resolved through discussion. An iterative approach will be taken with the data charting process and major revisions with rationale will be included in the final report. Critical appraisal of individual sources of evidence will not be completed as it is out of the scope of this review.

This scoping review has a number of potential impacts. Firstly, it will further elucidate to implementation researchers how ToC are described in the literature, what is meant by the term ToC, the value that ToCs bring to interventions, what is missing from these descriptions, and encourage consideration of how these descriptions could be improved. Secondly, for health service practitioners working within the field of child health service interventions, it will map the available evidence on how these interventions may use a ToC. Thirdly, this scoping review may encourage those in fields outside of child health service interventions to consider using a ToC as a program design, implementation, and evaluation tool.

### Study status

This scoping review will be conducted in early 2022.

## Data availability

### Underlying data

No underlying data are associated with this article. 

### Extended data

Open Science Framework: Using theory of change in child health service interventions: a scoping review protocol. ( Jones, 2022)

https://doi.org/10.17605/OSF.IO/5TPGM

This project contains the following extended data:

- Search strategies.docx
- Data extraction instrument.docx
- Theory of change diagram vs logic model examples.docx

Data are available under the terms of the Creative Commons Zero "No rights reserved" data waiver (CC0 1.0 Public domain dedication).

## References

[ref-1] Anderson AA : The Community Builder's Approach to Theory of Change: A Practical Guide to Theory Development.Aspen Institute Roundtable on Community Change.2006. Reference Source

[ref-2] Blamey A Mackenzie M : Theories of Change and Realistic Evaluation: Peas in a Pod or Apples and Oranges? *Evaluation.* 2007;13(4):439–455. 10.1177/1356389007082129

[ref-3] Breuer E Lee L De Silva M : Using theory of change to design and evaluate public health interventions: a systematic review. *Implement Sci.* 2016;11:63. 10.1186/s13012-016-0422-6 27153985 PMC4859947

[ref-4] Center for Theory of Change: What is Theory of Change? 2021. Reference Source

[ref-5] Chen HT Rossi PH : The Theory-Driven Approach to Validity. *Evaluation and Program Planning.* 1987;10(1):95–103. 10.1016/0149-7189(87)90025-5

[ref-6] Connell J Kubisch A : Applying a Theory of Change Approach to the Evaluation of Comprehensive Community Initiatives: Progress, Prospects, and Problems.In *New Approaches to Evaluat-ing Community Initiatives, Theory, Measurement, and Analysis*. Washington DC: Aspen Institute.1998;2. Reference Source

[ref-7] Connell J Kubisch A Schorr L : New Approaches to Evaluating Community Initiatives: Concepts, Methods and Contexts.New York: Aspen Institute.1995. Reference Source

[ref-8] De Silva MJ Breuer E Lee L : Theory of Change: a theory-driven approach to enhance the Medical Research Council's framework for complex interventions. *Trials.* 2014;15(1):267. 10.1186/1745-6215-15-267 24996765 PMC4227087

[ref-9] Hanson C Zamboni K Prabhakar V : Evaluation of the Safe Care, Saving Lives (SCSL) quality improvement collaborative for neonatal health in Telangana and Andhra Pradesh, India: a study protocol. *Glob Health Action.* 2019;12(1):1581466. 10.1080/16549716.2019.1581466 30849300 PMC6419630

[ref-10] Institute of Medicine Committee on Health Services Research: A Working Definition of Health Services Research.1994. Reference Source

[ref-11] Joanna Briggs Institute: Joanna Briggs Institute Reviewers’ Manual 2015. edition/Supplement. Retrieved from Australia:2015. Reference Source

[ref-12] Jones B : Using theory of change in child health service interventions: a scoping review protocol.2022. 10.17605/OSF.IO/5TPGM PMC8881691 35284641

[ref-13] Kumar P Chawla D Thukral A : Development of a quality improvement package for reducing sight-threatening retinopathy of prematurity. *Indian J Ophthalmol.* 2020;68(Suppl 1):S115–S120. 10.4103/ijo.IJO_2087_19 31937745 PMC7001182

[ref-14] LohrKN SteinwachsDM : Health services research: An evolving definition of the field. *Health Serv Res.* 2002;37(1):7–9. 11949927

[ref-15] Makowiecka K Marchant T Betemariam W : Characterising innovations in maternal and newborn health based on a common theory of change: lessons from developing and applying a characterisation framework in Nigeria, Ethiopia and India. *BMJ Glob Health.* 2019;4(4):e001405. 10.1136/bmjgh-2019-001405 31406587 PMC6666810

[ref-16] Peters MDJ Marnie C Tricco AC : Updated methodological guidance for the conduct of scoping reviews. *JBI Evid Synth.* 2020;18(10):2119–2126. 10.11124/JBIES-20-00167 33038124

[ref-17] Rogers P Petrosino, A Huebner T : Program Theory Evaluation: Practice, Promise, and Problems. *New Directions for Evaluation.* 2004;2000:5–13. Reference Source

[ref-18] Steinwachs D : Health-Services Research - Its Scope and Significance. *American Journal of Pharmaceutical Education.* 1991;55(3):274–278.

[ref-19] Taplin D Clark H : Theory of Change Basics A Primer on Theory of Change. Retrieved from New York:2012. Reference Source

[ref-20] Weiss CH : Nothing as Practical as Good Theory: Exploring Theory-Based Evaluation for Comprehensive Community-Based Initiatives for Children and Families.In *New Approaches to Evaluating Community Initiatives, Concepts, Methods and Contexts*. Washington, DC: Aspen Institute.1995;1. Reference Source

[ref-21] Weiss CH : How can theory-based evaluation make greater headway? *Evaluation Review.* 1997a;21(4):501–524. 10.1177/0193841X9702100405

[ref-22] Weiss CH : Theory-based evaluation: Past, present, and future. *New Dir Eval.* 1997b;1997(76):41–55. 10.1002/ev.1086

