# Supplementary material for: Using theory of change in child health service interventions: a scoping review protocol
Source: Wellcome Open Res. 2022 Jan 28;7:30. [Version 1] doi: 10.12688/wellcomeopenres.17553.1 (PMC8881691; doi:10.12688/wellcomeopenres.17553.1)
Supplement: Peer review history

# Peer review: Reviewer response for version 1

**DOI:** 10.21956/wellcomeopenres.19410.r48374

**Published:** 2022-02-25

**Authors:** Dale Barnhart

**Affiliations:** 1 Department of Global Health and Social Medicine, Harvard Medical School, Boston, MA, USA

This study protocol describes the process through which the authors will conduct a scoping review of the use of theories of change in child health service interventions. The author’s goals of understanding how theories of changes are developed and used in this context are well defined. The search strategy is well-defined, and the research team has thoughtfully considered how to account for mislabeled “theories of change” by proposing a clear definition for the term and including related terms in the search criteria. Data collection tools are provided; however, as this is a protocol for a future study, the data itself is not yet available.

Is the study design appropriate for the research question?

Yes

Is the rationale for, and objectives of, the study clearly described?

Yes

Are sufficient details of the methods provided to allow replication by others?

Yes

Are the datasets clearly presented in a useable and accessible format?

Not applicable

Reviewer Expertise:

implementation science; epidemiology

I confirm that I have read this submission and believe that I have an appropriate level of expertise to confirm that it is of an acceptable scientific standard.

# Peer review: Reviewer response for version 1

**DOI:** 10.21956/wellcomeopenres.19410.r48376

**Published:** 2022-02-18

**Authors:** Solange Mianda

**Affiliations:** 1 School of Public Health, University of Western Cape, Cape Town, South Africa; 2 CHESAI, Cape Town, South Africa

Thank you for the opportunity to review this paper

 I have read the paper with interest and I think the background idea is interesting.

 Child health is a growing global concern, and implementing and/or evaluating health service programmes with the promise to improve child health become imperative. While thinking of programmes that may provide better gains for child survival, developing a good theory of change (TOC) in the implementation and/or evaluation of such programmes could bear multiple benefits as enumerated by the authors. Hence, the need to better understand how TOC is defined (clarity of concept), developed and used (the object of this review protocol).

**Comments to the authors:**

- I have found that the rationale for, and the study were clearly described. I would like to subject that the heading ' ***objective'*** be included in the paper to guide the reader on the flow of the steps followed in the review. As it stands, the objective seems lost in the introduction.
- The sentence “ *This  scoping  review  is  intended  to  inform  organisations  working  in the  field  of  child  health  about  how  they  may  design  and  utilise  their  own  ToC*”, seems restrictive. There may be individuals e.g. students, public health practitioners, health promoters who may be interested in designing their own TOC for particular interventions and not be part of a given organization.
  If *“this  scoping  review may  encourage  those  in  fields  outside  of  child  health  service  interventions  to  consider  using  a  ToC  as  a  program  design,  implementation, and evaluation tool” *as stated by the authors on the potential impacts of this review, then the statement in the above paragraph should be corrected to expand to include a broader audience.
  In this sentence, I wonder whether the development, refining and use of TOC is done by researchers (designers of interventions, or interventions) “Ultimately,  the  objective  of  this  scoping review  is  to  map  the  evidence of?? how *child  health  service  interventions*  around  the  world  develop,  use,  and  refine  their  theory  of  change”
- The authors opted for the  JBI  methodology  for  scoping  reviews  (Joanna  Briggs  Institute,  2015;  Peters  et  al.,  2020)  although appropriate for the scope of study proposed by the authors, I did not find the rationale for this choice, given the existence of other approaches to scoping reviews.The authors have sufficiently provided details of the methods to allow replication by others.
- *Participant*
  The authors have stated that Health service interventions targeted at children and youth aged  0–19  years will be included in the review. What is the rationale for the choice of this age range? What is the definition of “child” in this review? The title is restricted to child, but the inclusion criteria is extended to youth. Providing a clear definition of who the authors consider as “child” needs to be provided to avoid confusion.
- 'Health service interventions' is a broad term and could be restrictive, Have the authors thought of interventions using terms as quality improvement or implementation? Many interventions make use of these terms and will not necessarily use intervention in the title, how would the authors account for articles that could be missed should these terms not be included in the search terms?

I look forward to seeing the end product, sounds very interesting, best wishes to the authors

Is the study design appropriate for the research question?

Yes

Is the rationale for, and objectives of, the study clearly described?

Yes

Are sufficient details of the methods provided to allow replication by others?

Yes

Are the datasets clearly presented in a useable and accessible format?

Yes

Reviewer Expertise:

Health systems governance, health policy and systems research, maternal and child health, implementation research, health systems strengthening, programme evaluation, evidence synthesis

I confirm that I have read this submission and believe that I have an appropriate level of expertise to confirm that it is of an acceptable scientific standard.
